# Single-cell transcriptomic analysis of cutaneous squamous cell carcinoma identifies an NR1H3-associated angiogenic macrophage state

**DOI:** 10.3389/fimmu.2026.1837479

**Published:** 2026-07-30

**Authors:** Yong He, Ting Tian, Liming Li, Leqi Qian, Tian Tian, Xiaoke Wang, Yuancheng Li, Mingjun Jiang

**Affiliations:** Hospital for Skin Diseases, Institute of Dermatology, Chinese Academy of Medical Sciences & Peking Union Medical College, Nanjing, Jiangsu, China

**Keywords:** cutaneous squamous cell carcinoma, intercellular communication, NR1H3, single-cell transcriptomics, tumor microenvironment, tumor-associated macrophages

## Abstract

**Introduction:**

Cutaneous squamous cell carcinoma (cSCC) is a common keratinocyte-derived malignancy whose progression is strongly influenced by the tumor microenvironment. Although recent studies have begun to describe cellular diversity in squamous cell carcinomas, the regulatory architecture coordinating epithelial, immune, and stromal interactions in cSCC remains incompletely understood.

**Methods:**

We performed single-cell RNA sequencing on matched tumor tissues, adjacent normal skin, and peripheral blood from patients with cSCC to construct an integrated cellular landscape of the tumor ecosystem. Lineage trajectory analysis, transcription factor activity inference, and ligand–receptor modeling were used to characterize cellular differentiation, regulatory programs, and intercellular communication within the cSCC microenvironment.

**Results:**

Analysis of 80,736 single cells revealed extensive heterogeneity across epithelial, immune, and stromal compartments. Trajectory analysis of the myeloid lineage suggested a tumor-associated differentiation continuum extending from circulating monocytes toward a specialized angiogenic macrophage state characterized by transcriptional programs associated with extracellular matrix remodeling and immunoregulatory signaling. Concurrently, malignant keratinocyte populations displayed inferred copy-number alterations and activation of invasive transcriptional programs, accompanied by the emergence of distinct cancer-associated fibroblast subsets associated with stromal remodeling. Ligand–receptor modeling further identified extensive predicted communication networks linking macrophages, fibroblasts, and malignant epithelial cells within the tumor niche. Furthermore, transcription factor activity inference highlighted the nuclear receptor *NR1H3* as a candidate regulator associated with the angiogenic macrophage program, and the *NR1H3*-associated transcriptional signature correlated with poor survival across multiple squamous cell carcinoma cohorts.

**Discussion:**

These findings define the cellular organization and molecular programs that shape the cSCC microenvironment and provide insights into the coordinated immune, stromal, and epithelial interactions involved in cSCC progression.

## 1 Introduction

Cutaneous squamous cell carcinoma (cSCC) is one of the most common keratinocyte-derived malignancies and represents a major proportion of non-melanoma skin cancers worldwide (1, 2). While early-stage cSCC is largely curable through surgical excision, the management of advanced or metastatic disease remains a major clinical challenge (3). The advent of immune checkpoint blockade, particularly inhibitors targeting the PD-1/PD-L1 axis, has emerged as an effective therapeutic strategy for advanced cSCC (4). However, clinical responses remain heterogeneous, and a substantial fraction of patients exhibit primary or acquired resistance to immunotherapy (5), highlighting the critical influence of the tumor microenvironment in regulating disease progression and treatment response.

The cSCC microenvironment consists of a complex multicellular ecosystem where interactions among malignant keratinocytes, stromal fibroblasts, and diverse immune populations collectively drive immune evasion and therapeutic resistance (6, 7). Among these components, myeloid cells represent a major immune population in many solid tumors and play essential roles in shaping tumor progression. In particular, tumor-associated macrophages (TAMs) display remarkable functional plasticity and can promote tumor growth through mechanisms including angiogenesis, extracellular matrix remodeling, and suppression of cytotoxic T-cell responses (8, 9). In parallel, stromal fibroblasts can differentiate into specialized cancer-associated fibroblast (CAF) subsets that regulate inflammatory signaling, matrix deposition, and tissue remodeling, thereby contributing to the formation of tumor-supportive niches (10, 11).

Recent advances in single-cell RNA sequencing (scRNA-seq) have enabled high-resolution dissection of tumor ecosystems and revealed extensive cellular heterogeneity across epithelial, immune, and stromal compartments in cSCC. Single-cell studies of squamous cell carcinomas have begun to uncover diverse malignant keratinocyte states and complex interactions between tumor cells and the surrounding microenvironment (12–14). Despite these advances, several key aspects of the cSCC microenvironment remain incompletely understood. In particular, the developmental relationships among myeloid cell populations, the heterogeneity and evolutionary trajectories of fibroblast subsets, and the global intercellular communication networks that coordinate tumor–stroma–immune interactions have not been systematically characterized.

In this study, we performed single-cell transcriptomic profiling of matched tumor tissues, adjacent normal skin, and peripheral blood samples from patients with cSCC. By constructing an integrated cellular landscape of the cSCC microenvironment, we systematically characterized the heterogeneity of epithelial, stromal, and immune compartments and reconstructed lineage relationships within the myeloid and fibroblast populations. Furthermore, we integrated trajectory inference and cell–cell communication analyses to investigate the regulatory interactions among these cellular components. This work provides a comprehensive framework for understanding the cellular organization and multicellular communication networks that shape the cSCC tumor ecosystem.

## 2 Materials and methods

### 2.1 Patient cohort, ethical approval, and tissue acquisition

Fresh primary human cSCC specimens, alongside patient-matched adjacent macroscopically normal skin and peripheral blood, were surgically procured from inpatients admitted to the Hospital for Skin Diseases, Institute of Dermatology, Chinese Academy of Medical Sciences & Peking Union Medical College. Prior to inclusion in this transcriptomic study, the histopathological diagnosis of cSCC for every individual case was rigorously evaluated and corroborated by board-certified dermatopathologists. Clinical and pathological characteristics of the included patients, including tumor differentiation status, are summarized in **Supplementary Table 1**. The study design was reviewed and officially approved by the Institutional Ethics Committee of the Hospital for Skin Diseases, Chinese Academy of Medical Sciences. Written informed consent was obtained from all participating patients prior to surgical intervention.

### 2.2 Tissue dissociation and single-cell suspension preparation

Immediately post-excision, solid tissue specimens were immersed in a commercial tissue preservation solution and transported to the laboratory under strict cold-chain conditions (4°C). Tissues were extensively washed with chilled phosphate-buffered saline (PBS), mechanically minced into microscopic fragments, and subjected to enzymatic digestion utilizing the tumor dissociation kit (Miltenyi Biotec, Germany) according to the manufacturer’s optimized protocols. The resulting cellular homogenates were sequentially passed through 40 μm sterile cell strainers. The filtered cell suspensions were centrifuged and resuspended in PBS containing 0.04% bovine serum albumin. Only single-cell suspensions demonstrating robust viability (> 80%) via trypan blue exclusion were authorized for immediate loading onto the 10x Genomics Chromium Controller platform for subsequent microfluidic droplet generation, barcoding, and scRNA-seq library construction.

### 2.3 scRNA-seq data processing, quality control, and integration

Raw sequencing reads were aligned to the human reference genome (GRCh38) and quantified. To ensure high-quality downstream analysis, rigorous quality control (QC) was applied to the initial dataset using the Seurat R package (15). Cells were retained only if they met the following stringent criteria: the number of detected genes was between 200 and 6, 000, the proportion of mitochondrial transcripts was less than 20%, and the proportion of ribosomal transcripts was greater than 5%. Putative doublets were systematically identified and removed utilizing the DoubletFinder package (16). Following QC, the filtered data were log-normalized. To integrate multiple matched samples and mitigate inter-patient batch effects, the Harmony algorithm was employed (17). Principal Component Analysis (PCA) and unsupervised Louvain clustering were performed on highly variable genes, and Uniform Manifold Approximation and Projection (UMAP) was utilized for two-dimensional visualization.

### 2.4 High-resolution subclustering and malignancy inference

Major cell lineages initially identified in the global landscape were computationally extracted for secondary, high-resolution subclustering. Each extracted subset underwent iterations of normalization, scaling, PCA, and clustering to delineate specific functional states. Differentially expressed marker genes used for cell lineage and subpopulation annotation are provided in **Supplementary Table 2**. To specifically identify malignant cells within the keratinocyte compartment, large-scale chromosomal copy number variations (CNVs) were inferred using inferCNV (18). Annotated T and NK cells were utilized as the diploid reference. A 6-state Hidden Markov Model (HMM) was utilized to predict CNV states, and the overall CNV burden was quantified to validate the malignant identities of Tumor-Specific Keratinocytes (TSKs). For sample-stratified assessment, CNV scores were calculated for individual keratinocytes based on the mean absolute inferred CNV signal and then visualized across keratinocyte subclusters within each patient.

### 2.5 Pseudotime trajectory inference

To reconstruct the dynamic developmental progressions within the tumor microenvironment, single-cell pseudotime trajectory analysis was conducted using the Monocle 3 package (19). For the myeloid compartment, the trajectory root was assigned to the classical monocyte (cMono) cluster to model the transition towards terminally differentiated tissue-resident TAMs, specifically delineating the distinct developmental bifurcations into angiogenic TAM (Angio-TAM) and inflammatory TAM (Inflam-TAM) subpopulations. To evaluate systemic-to-local myeloid remodeling, myeloid pseudotime values were further stratified according to tissue origin, including peripheral blood, paratumor, and tumor compartments. Similarly, the developmental continuum of the fibroblast-to-CAF transition was reconstructed by anchoring the trajectory root at the steady-state fibroblast cluster, allowing for the visualization of the differentiation timeline toward inflammatory CAF (iCAF) and matrix CAF (mCAF) terminal states.

### 2.6 Intercellular communication analysis

Cell-cell communication networks within the cSCC ecosystem were inferred using the CellChat R package (20). Communication probability was calculated, and statistically significant interactions were identified using a permutation test (*p* < 0.05). The contribution of specific ligand-receptor pairs and major signaling pathways to the immunosuppressive niche was evaluated. The hierarchical organization and directionality of the signaling networks were visualized using heatmaps, bubble plots, and chord diagrams.

### 2.7 Multiplex immunofluorescence staining

Multiplex immunofluorescence staining was performed on formalin-fixed paraffin-embedded cSCC tissue sections. Briefly, 4-μm sections were baked, deparaffinized in xylene, and rehydrated through graded ethanol. Antigen retrieval was performed using citrate buffer under microwave heating. After blocking, sections were incubated with primary antibodies against CD163 (Proteintech, Cat No. 16646-1-AP), SPP1 (Proteintech, Cat No. 22952-1-AP), and α-SMA (Proteintech, Cat No. 14395-1-AP), followed by HRP-conjugated secondary antibodies. Fluorescent signals were developed using tyramide signal amplification (TSA) reagents (Servicebio, G1236) according to the manufacturer’s instructions. For multiplex staining, sequential rounds of antibody incubation, TSA detection, and microwave stripping were performed for each marker. Nuclei were counterstained with DAPI (Servicebio, G1012), and slides were mounted with antifade mounting medium. Fluorescence images were acquired using a fluorescence microscope to evaluate the spatial distribution of CD163^+^SPP1^+^ macrophages and α-SMA^+^ CAF in tumor and paratumoral regions.

### 2.8 Single-cell regulatory network and virtual knockout

Single-cell transcription factor activities were inferred using the decoupleR R package coupled with the DoRothEA prior knowledge network (21, 22). Transcription factor enrichment scores were calculated utilizing the Univariate Linear Model (ULM) to identify subpopulation-specific master regulators. To evaluate the downstream regulatory effects of *NR1H3*, an *in silico* virtual knockout was performed utilizing the scTenifoldKnk R package (23). We simulated the knockout by computationally setting the *NR1H3* expression matrix to zero. By comparing the perturbed regulatory network against the wild-type baseline, transcriptomic shifts were quantified as perturbation distances to identify and rank the significantly affected downstream target genes.

### 2.9 Functional enrichment and metabolic profiling

Pathway activity across different subclusters was estimated using Gene Set Variation Analysis (GSVA) with MSigDB Hallmark gene sets (24, 25). Functional module scores for specific biological processes were calculated using the AddModuleScore function in Seurat (26). For T/NK cell functional remodeling analysis, module scores representing cytotoxicity, checkpoint-related activity, tissue residency, and Treg/immunoregulatory programs were calculated and compared across peripheral blood, paratumor, and tumor compartments. Differentially expressed genes and targets identified from the *in silico* perturbation were subjected to GO and KEGG enrichment analyses to identify over-represented biological processes.

### 2.10 Survival analysis across SCC cohorts

The prognostic significance of the *NR1H3*-driven Angio-TAM regulatory axis was evaluated across multiple squamous cell carcinoma (SCC) cohorts utilizing the Gene Expression Profiling Interactive Analysis 2 (GEPIA2) web server (27). A customized 10-gene prognostic signature was constructed to evaluate the clinical relevance of the Angio-TAM subpopulation. This signature strictly consists of the identified master transcriptional regulator (*NR1H3*), two core Angio-TAM marker genes (*SPP1* and *APOE*), and seven critical effector genes (*CXCL3*, *CCL20*, *CXCL8*, *CCL3L1*, *EREG*, *PHLDA1*, and *CD9*). These seven effectors were directly selected by intersecting the significantly upregulated genes in the Angio-TAM cluster with the top perturbed downstream targets identified from the *NR1H3 in silico* knockout. Clinical metadata and bulk transcriptomic data from independent The Cancer Genome Atlas (TCGA) cohorts for head and neck (HNSC), lung (LUSC), and cervical (CESC) squamous cell carcinomas were analyzed (28). For each patient, an overall expression score for the 10-gene signature was calculated based on the expression levels of the genes included in the panel. Within each TCGA cohort, patients were then stratified into high- and low-expression groups using the median signature score as the cutoff. Kaplan-Meier overall survival (OS) curves were generated, and statistical significance was assessed via the log-rank test, accompanied by hazard ratio (HR) calculations.

### 2.11 External cohort validation and reference mapping

To rigorously validate the robustness of the identified Angio-TAM phenotype and spatial distribution, an independent single-cell cSCC dataset (GSE144240) was retrieved and processed (12). We utilized the Canonical Correlation Analysis (CCA) algorithm to map our well-annotated myeloid subset labels onto the external query dataset. Specifically, the FindTransferAnchors function was applied using CCA, followed by TransferData to compute prediction scores and assign validated cell type identities (29). Key functional module scores and core Angio-TAM signature expressions were subsequently recalculated in this validation cohort to verify phenotypic conservation.

### 2.12 *In vitro* validation of the NR1H3-associated macrophage program

A431-conditioned medium was collected after culturing A431 cells in medium containing 1% FBS for 24 h, followed by centrifugation and filtration. THP-1-derived macrophage-like cells were transfected with negative-control siRNA or siRNA targeting *NR1H3* and treated with 50% A431-conditioned medium for 24 h. *NR1H3* and *APOE* expression levels were measured by RT-qPCR using *ACTB* as the internal control and calculated using the 2^−^ΔΔCt method. Primer and siRNA sequences are listed in **Supplementary Table 3**. For the invasion assay, conditioned supernatants from the indicated macrophage-like cell treatment groups were collected and placed in the lower chamber together with complete medium. A431 cells were seeded into the upper chamber of Transwell inserts, and invaded cells were stained, imaged, and quantified after incubation.

## 3 Results

### 3.1 A single-cell transcriptomic landscape of the cSCC microenvironment

**Figure 1:**
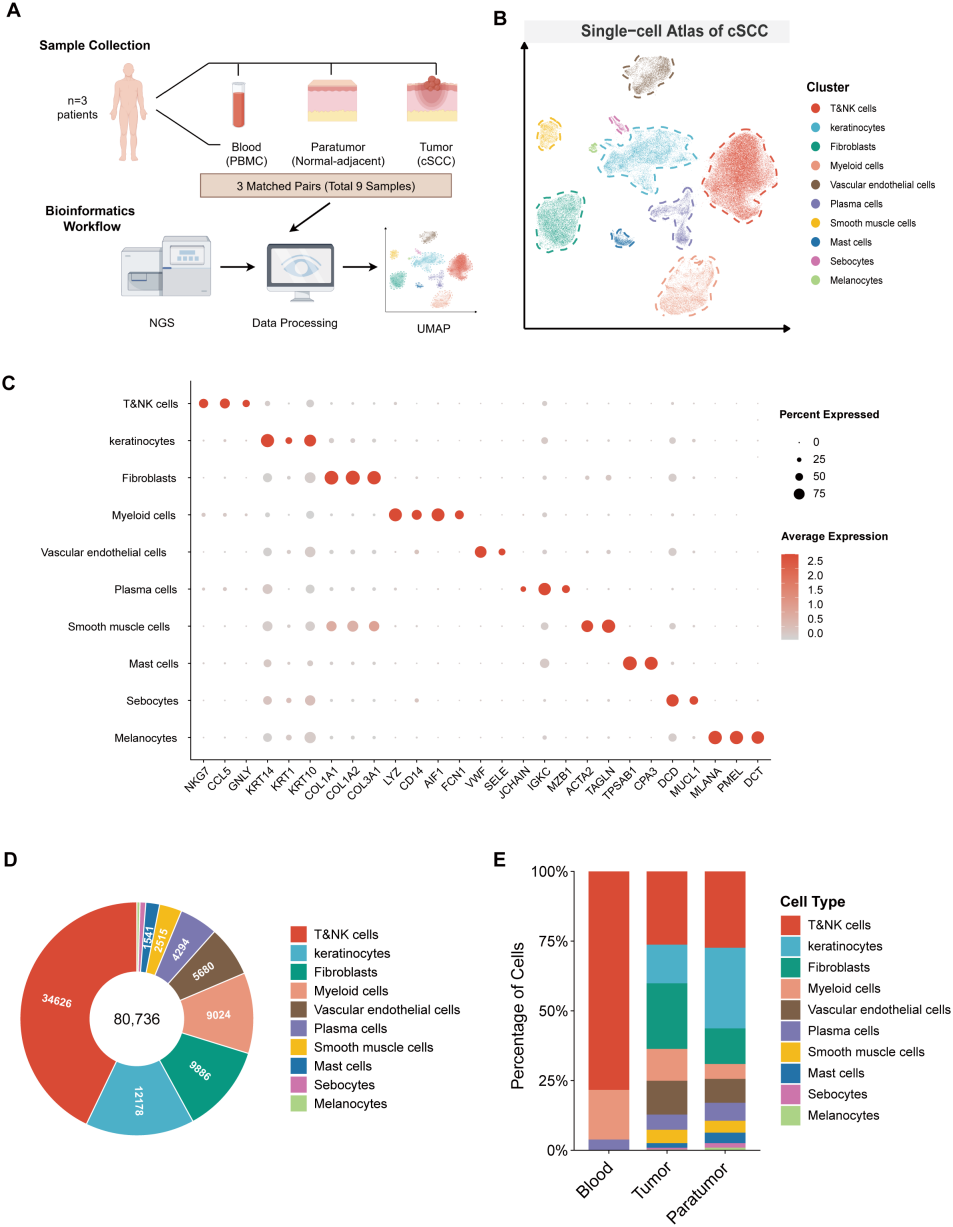
Single-cell transcriptional landscape of cutaneous squamous cell carcinoma (cSCC). **(A)** Schematic representation of the experimental design and single-cell RNA sequencing workflow. Matched peripheral blood, tumor, and paratumor tissues were collected from three cSCC patients. **(B)** UMAP plot of the global single-cell landscape of cSCC, encompassing 80, 736 cells. Each individual point represents one cell, and the color of each point corresponds to the annotated cell identity. **(C)** Dot plot illustrating the expression of canonical marker genes used to define the major cell lineages. The dot size represents the percentage of cells expressing the gene, and the color intensity indicates the average expression level. **(D)** Donut chart detailing the total number and specific proportion of cells captured for each major cell type in the integrated dataset. **(E)** Stacked bar plot showing the proportional distribution of the identified cell types across different tissue origins (blood, tumor, and paratumor).

To comprehensively characterize the cellular ecosystem of cSCC, we performed scRNA-seq on matched tumor, adjacent normal skin, and peripheral blood samples from three patients (**Figure 1A**). Following quality control, our final integrated landscape comprised a robust dataset of 80, 736 high-quality cells.

UMAP dimensionality reduction delineated 10 transcriptionally distinct cell lineages (**Figure 1B**). These cells were annotated based on the expression of established canonical markers, such as *NKG7* for T and NK cells, *KRT14* for keratinocytes, *COL1A1* for fibroblasts, and *LYZ* for myeloid cells (**Figure 1C**; **Supplementary Figure 1A**) (30). Other identified subsets included vascular endothelial cells, plasma cells, smooth muscle cells, mast cells, sebocytes, and melanocytes. Hierarchical clustering of the global correlation matrix further validated these assignments, correctly segregating stromal, epithelial, and immune lineages into their respective developmental clades (**Supplementary Figure 1B**).

Quantitative assessment of the cellular composition revealed that the microenvironment was predominantly composed of T/NK cells, followed by keratinocytes, fibroblasts, and myeloid cells (**Figure 1D**). Finally, an evaluation of tissue distribution highlighted distinct compositional shifts driven by the malignant state. While peripheral blood was exclusively populated by circulating immune cells, both tumor and paratumor tissues exhibited a complex, multicellular interacting network (**Figure 1E**). Despite anticipated inter-patient variability in specific subset proportions, the core cellular composition remained biologically consistent across biological replicates, suggesting a universal pattern of tumor microenvironment remodeling in cSCC (**Supplementary Figure 1C**).

### 3.2 High-resolution subclustering reveals a tumor-induced monocytic trajectory that programs the Angio-TAM state

**Figure 2:**
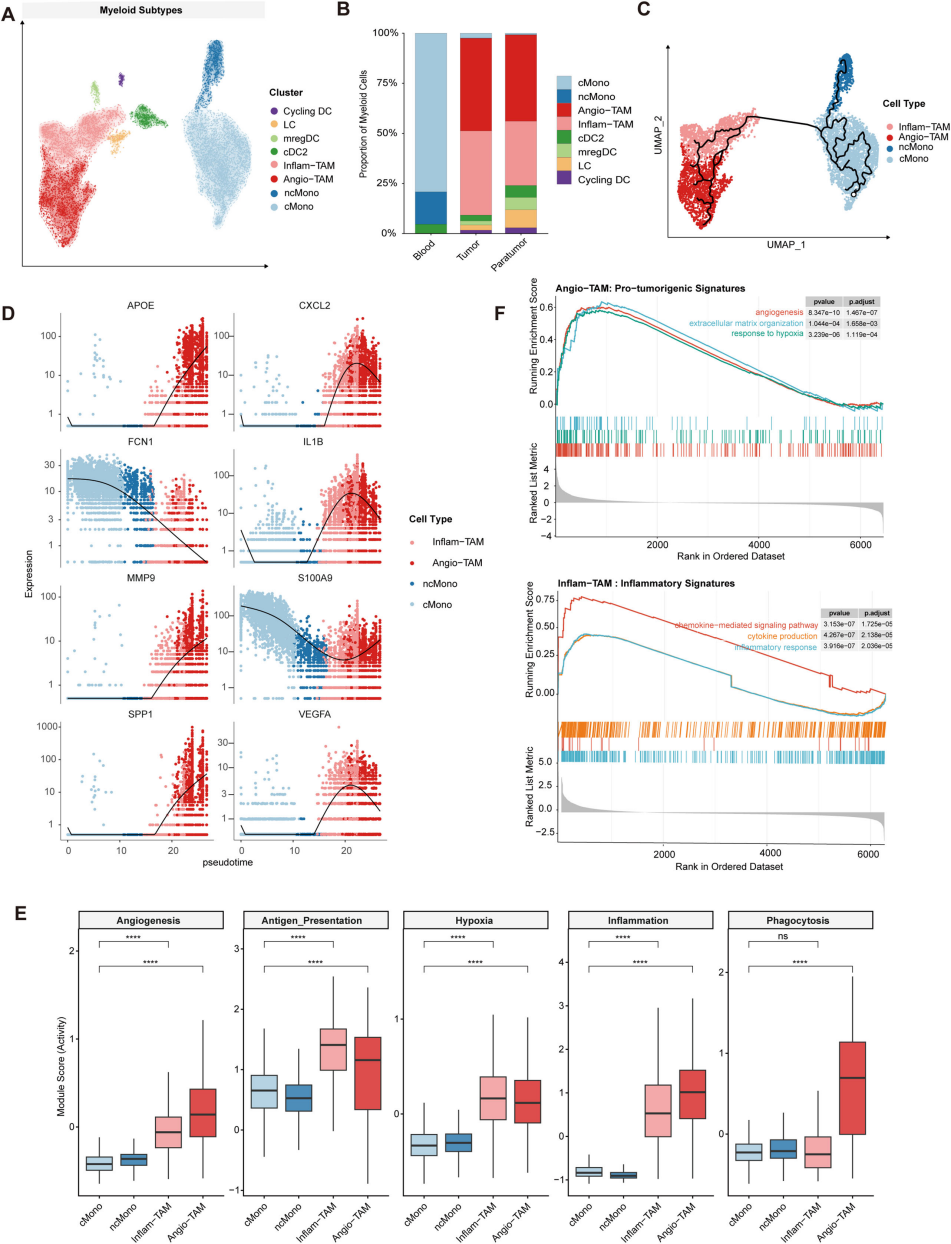
High-resolution transcriptomic landscape and developmental trajectory of myeloid cells in cSCC. **(A)** UMAP plot revealing the subclustering of the myeloid lineage into eight distinct subpopulations. Each individual point represents one cell, and the color of each point corresponds to the annotated myeloid identity. **(B)** Stacked bar plot illustrating the proportional distribution of each myeloid subset across blood, tumor, and paratumor tissues, highlighting the tumor-specific enrichment of Angio-TAMs. **(C)** Pseudotime trajectory projected onto the UMAP embedding, reconstructing the developmental progression from circulating monocytes (cMono, ncMono) to tissue-resident macrophages (Inflam-TAM, Angio-TAM). The black line indicates the principal graph of the inferred developmental lineage. **(D)** Scatter plots depicting the gene expression dynamics of representative monocytic (*FCN1*, *S100A9*), inflammatory (*IL1B*, *CXCL2*), and pro-angiogenic/remodeling (*APOE*, *MMP9*, *SPP1*, *VEGFA*) markers along the pseudotime continuum. **(E)** Box plots comparing the activity scores of specific functional modules across monocytic and TAM subsets (****p < 0.0001; ns, not significant). **(F)** Gene Set Enrichment Analysis (GSEA) plots showcasing the distinct functional signatures enriched in Angio-TAMs (top, pro-tumorigenic and hypoxic signatures) and Inflam-TAMs (bottom, inflammatory and chemokine signaling).

To further delineate the heterogeneity of the myeloid compartment within the cSCC microenvironment, we performed high-resolution subclustering of this lineage. The analysis identified eight distinct myeloid subsets, including two monocyte states (cMono, non-classical monocytes [ncMono]), two macrophage states (Inflam-TAM, Angio-TAM), and four dendritic cell subsets: type 2 conventional dendritic cells (cDC2), mature regulatory dendritic cells (mregDC), Langerhans cells (LC), and cycling dendritic cells (Cycling DC) (**Figure 2A**). The identities of these subpopulations were confirmed by their specific transcriptional profiles, as visualized in the comprehensive marker gene dot plot (**Supplementary Figure 2A**). Tissue distribution analysis revealed that circulating monocytes were predominantly restricted to the blood, whereas TAMs heavily infiltrated the solid tissues. Compositional analysis across tissue origins (**Figure 2B**) demonstrated that Angio-TAMs populated both paratumor and tumor tissues and represented a prominent myeloid population within the cSCC microenvironment.

To reconstruct the developmental progression from blood monocytes to tissue-resident TAMs, we performed pseudotime trajectory analysis. The inferred trajectory originated from circulating monocytes (cMono and ncMono) and bifurcated toward the terminally differentiated Inflam-TAM and Angio-TAM states (**Figure 2C**). Stratification of myeloid pseudotime by tissue origin further showed that blood-derived myeloid cells were enriched at early pseudotime states, whereas paratumor- and tumor-derived myeloid cells occupied later pseudotime states, supporting a systemic-to-local monocyte-to-TAM transition in cSCC (**Supplementary Figure 2B**). Tracking gene expression dynamics along this pseudotime continuum revealed a progressive loss of monocytic markers (e.g., *FCN1*, *S100A9*) alongside a dramatic, late-stage increase in the expression of Angio-TAM-defining functional effectors, such as *SPP1*, *APOE*, *VEGFA*, and *MMP9* (**Figure 2D**; **Supplementary Figure 2C**). In contrast, inflammatory markers like *IL1B* and *CXCL2* peaked during the transition and terminal phases of the Inflam-TAM branch, suggesting an alternative activation state (31).

Functional module scoring revealed that terminally differentiated Angio-TAMs exhibited the highest activity scores in angiogenesis and phagocytosis, while the transitional Inflam-TAMs scored highest in general inflammation and antigen presentation (**Figure 2E**). Gene Set Enrichment Analysis (GSEA) supported these functional distinctions. In contrast to Inflam-TAMs, which primarily exhibited enrichment for canonical inflammatory responses and chemokine signaling, GSEA demonstrated that Angio-TAMs were specifically enriched for signatures related to angiogenesis, extracellular matrix (ECM) organization, and response to hypoxia (**Figure 2F**). To systematically dissect the signaling cascades driving this terminal phenotype, we performed Gene Ontology (GO), Kyoto Encyclopedia of Genes and Genomes (KEGG), and Reactome pathway analyses (**Supplementary Figures 2D–F**). These pathway analyses further highlighted the involvement of Angio-TAMs in chemokine-mediated signaling pathways, leukocyte chemotaxis, and cytokine-cytokine receptor interactions. Functionally, these features suggest that Angio-TAMs may support cSCC progression by promoting tumor vascularization, facilitating extracellular matrix remodeling, and shaping a pro-tumorigenic immune niche. Together, these findings characterize Angio-TAMs as a specialized macrophage state that develops from recruited blood monocytes and exhibits transcriptional programs associated with angiogenesis, extracellular matrix remodeling, and chemokine/cytokine-mediated immune niche conditioning.

### 3.3 Malignant keratinocyte transformation synergizes with fibroblast-to-CAF transition dynamics

**Figure 3:**
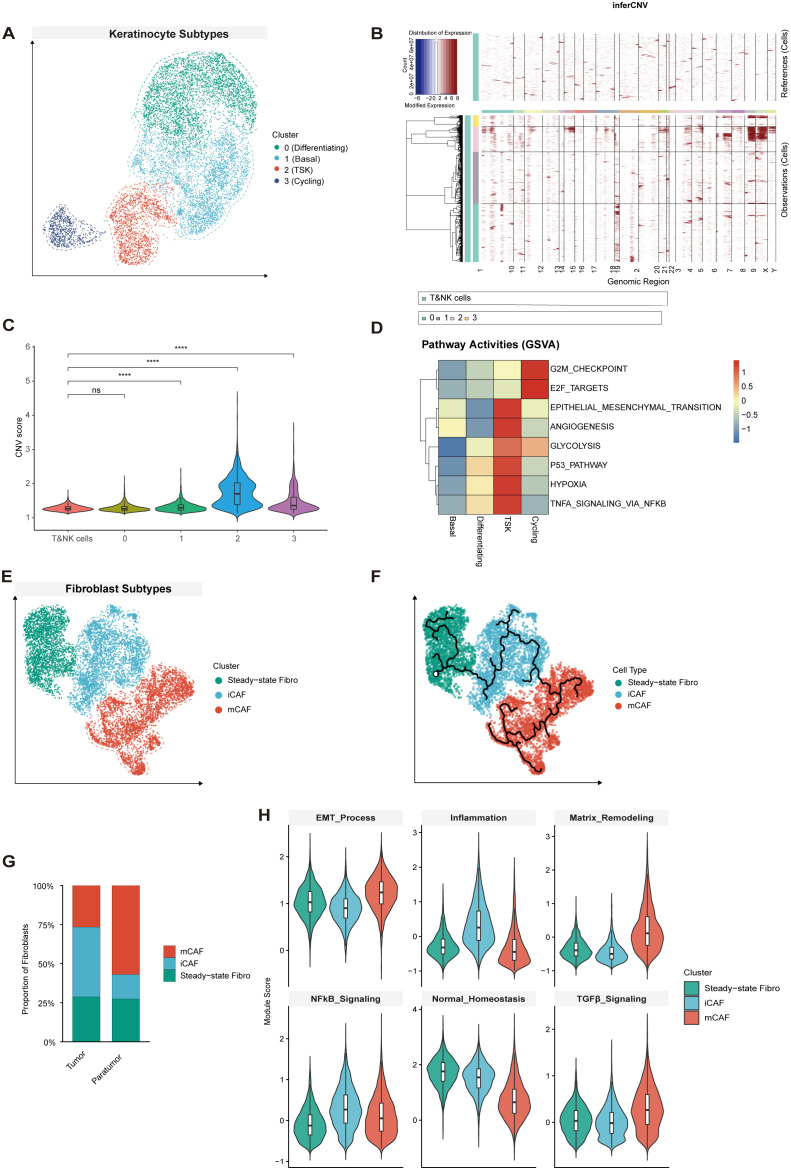
Malignant transformation of keratinocytes and the co-evolutionary trajectory of cancer-associated fibroblasts (CAFs). **(A)** UMAP plot depicting the subclustering of keratinocytes into four functional states: 0 (Differentiating), 1 (Basal), 2 (Tumor-Specific Keratinocytes) (TSK), and 3 (Cycling cells). **(B)** Heatmap illustrating large-scale chromosomal copy number variations (CNVs) across keratinocyte subsets inferred by inferCNV, with T and NK cells serving as the diploid reference. Red and blue indicate genomic amplifications and deletions, respectively. **(C)** Violin plot quantifying the overall CNV burden across the reference T&NK cells and keratinocyte subpopulations (****p< 0.0001; ns, not significant). **(D)** Heatmap of Gene Set Variation Analysis (GSVA) scores, highlighting the differential activation of canonical hallmark pathways across keratinocyte subsets. **(E)** UMAP plot revealing the heterogeneity of the fibroblast compartment, partitioned into Steady-state Fibro, inflammatory CAFs (iCAF), and matrix CAFs (mCAF). **(F)** Pseudotime trajectory projected onto the UMAP space, reconstructing the continuous developmental lineage from Steady-state Fibroblasts to the iCAF and mCAF terminal states. The solid black line indicates the principal evolutionary graph. **(G)** Stacked bar plot showing the proportional distribution of fibroblast subsets between tumor and paratumor tissues. **(H)** Violin plots comparing the specific functional module activities among the three fibroblast subsets.

To comprehensively characterize the malignant ecosystem of cSCC, we analyzed the epithelial and stromal populations at the single-cell level. Unsupervised clustering of keratinocytes identified four distinct functional states: Basal, Differentiating, Cycling, and a prominent TSK population (**Figure 3A**). The identities of these subsets were identified by the specific expression of canonical markers, such as *KRT5* for Basal cells, *KRT1* and *KRT10* for Differentiating cells, *MKI67* for Cycling cells, and *MMP1* and *LAMC2* for TSKs (**Supplementary Figure 3A**) (12). To confirm their neoplastic nature, we performed inferCNV analysis, which revealed that TSKs and Cycling keratinocytes had widespread chromosomal copy number variations (CNVs) compared to the diploid reference subsets (**Figure 3B**). Quantitative assessment corroborated that TSKs exhibited the highest degree of genomic instability, significantly exceeding all other epithelial clusters (**Figure 3C**; *p*< 0.0001). To assess whether this CNV pattern was driven by individual samples, we further stratified CNV scores by patient and observed elevated CNV burden in malignant keratinocyte clusters across individual cSCC samples, although the magnitude varied among patients (**Supplementary Figure 3B**). Furthermore, functional profiling via GSVA and GSEA illustrated that TSKs were uniquely enriched in epithelial-mesenchymal transition (EMT), hypoxia, and TNF-α signaling, while concurrently downregulating oxidative phosphorylation (**Figure 3D**; **Supplementary Figure 3C**), indicating a metabolic shift toward an invasive phenotype.

The aggressive phenotype of TSKs was accompanied by extensive stromal remodeling within the tumor microenvironment. High-resolution subclustering of the fibroblast compartment delineated normal steady-state fibroblasts and two specialized CAF states: inflammatory CAFs (iCAFs) and matrix CAFs (mCAFs) (**Figure 3E**; **Supplementary Figure 3D**) (32). Trajectory inference reconstructed a continuous differentiation continuum originating from homeostatic steady-state fibroblasts that bifurcated toward distinct iCAF and mCAF terminal states (**Figure 3F**). Pseudotime quantification confirmed mCAFs as the most advanced differentiation endpoint (**Supplementary Figures 3E, F**).

Interestingly, compositional analysis revealed a preferential expansion of mCAFs within the paratumoral tissues rather than the tumor core (**Figure 3G**). Consistent with this peripheral enrichment, functional module scoring demonstrated that mCAFs were highly specialized for ECM remodeling and TGF-β signaling, whereas iCAFs were characterized by a robust inflammatory signature (**Figure 3H**). These findings suggest that mCAFs may contribute to ECM remodeling in the tumor-adjacent stromal compartment, potentially supporting the formation of a desmoplastic microenvironment associated with malignant invasion (32, 33). Together, these findings characterize a functionally coupled epithelial–stromal ecosystem in cSCC, wherein malignant epithelial transformation is accompanied by the programmatic maturation of pro-tumorigenic CAF subsets.

### 3.4 Multicellular communication analysis uncovers fibroblast-mediated niche remodeling

**Figure 4:**
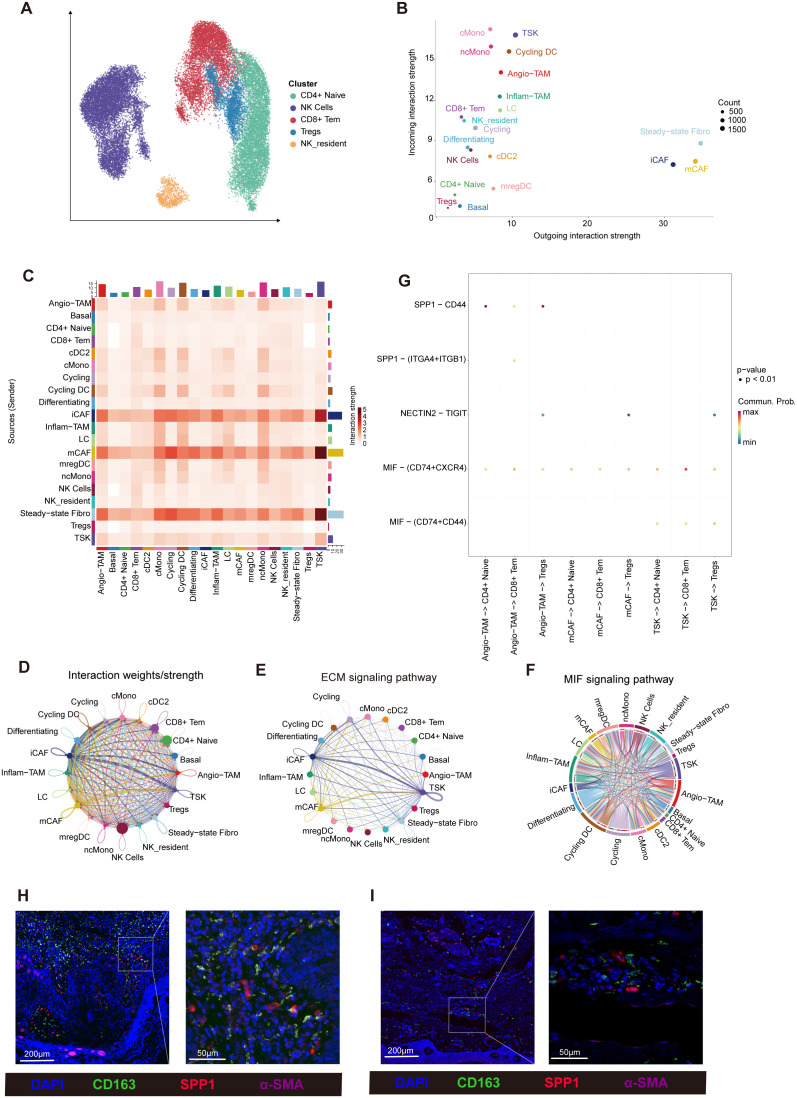
Multicellular communication landscape and immunosuppressive niche remodeling in cSCC. **(A)** UMAP plot depicting the subclustering of the T&NK cells into five distinct subpopulations: CD4^+^ Naive, NK Cells, CD8^+^ Tem, Tregs, and NK_resident cells. **(B)** Scatter plot evaluating the incoming and outgoing interaction strengths of all identified cell types, highlighting mCAFs and iCAFs as primary signal senders, and TSKs and Angio-TAMs as major signal receivers. **(C)** Heatmap quantifying the overarching cell-cell interaction strengths, with the y-axis representing the signal sources (Senders) and the x-axis representing the targets (Receivers). **(D)** Global network plot visualizing the overall interaction weights/strength among all cellular subsets in the tumor microenvironment. Line thickness correlates with interaction strength. **(E)** Network plot specifically highlighting the structural intercellular crosstalk mediated by the extracellular matrix (ECM) signaling pathway. **(F)** Chord diagram detailing the complex, directional intercellular communication driven by the MIF signaling pathway across the cSCC ecosystem. **(G)** Bubble plot demonstrating key immunosuppressive ligand-receptor pairs mediating crosstalk from stromal and myeloid cells to malignant TSKs and T cells. The dot size represents the statistical significance (*p*-value), and the color gradient indicates the communication probability. **(H-I)** Representative multiplex immunofluorescence images of matched cSCC tumor **(H)** and paratumoral **(I)** tissues. The sections were co-stained for DAPI (blue, nuclei), CD163 (green, macrophages), SPP1 (red, Angio-TAM marker), and α-SMA (pink, CAFs). The right panels show magnified views of the white boxed regions in the left panels.

To systematically resolve the intercellular regulatory network within the cSCC ecosystem, we employed CellChat to model cell-cell communications among the epithelial, stromal, and immune compartments. Unsupervised clustering of the T&NK cells initially identified five major distinct subsets, including CD8^+^ Tem, Tregs, and NK cells, each defined by canonical lineage markers (**Figure 4A**; **Supplementary Figure 4A**). To assess functional remodeling of T&NK cells across systemic and local compartments, we compared module scores among peripheral blood, paratumor, and tumor samples. Blood-derived T&NK cells exhibited higher cytotoxicity scores, whereas tissue-associated T&NK cells showed reduced cytotoxicity and relatively increased checkpoint-related, tissue-residency, and Treg-regulatory signatures (**Supplementary Figure 4B**). These compartment-associated changes suggest that local cSCC tissues may provide an immunosuppressive milieu characterized by attenuated cytotoxic activity and enhanced regulatory/checkpoint-associated T&NK programs. Scatter plot analysis of global interaction strengths identified mCAFs and iCAFs as the predominant signaling sources, whereas TSKs and selected myeloid subsets, particularly Angio-TAMs, emerged as major signal receivers (**Figure 4B**). Heatmap quantification of interaction weights further corroborated that CAFs established robust communication axes with the malignant TSK cluster (**Figure 4C**).

Global signaling analysis highlighted a dense and complex interaction network, with CAFs occupying a central hub position (**Figure 4D**; **Supplementary Figure 4C**). Notably, we identified a highly active ECM signaling pathway characterized by intense crosstalk between CAFs and TSKs (**Figure 4E**; **Supplementary Figure 4D**). This network was mainly characterized by collagen-mediated interactions, suggesting that CAF subsets may actively remodel the stromal architecture to facilitate the invasive progression of malignant TSKs (34). Concurrently, analysis of the MIF signaling pathway demonstrated that TSKs were involved as an important signaling source within a broad MIF-mediated communication network, establishing extensive communication networks with diverse downstream targets, including both myeloid and lymphoid immune compartments (**Figure 4F**).

To investigate cell–cell signaling architecture associated with this immune-evasive microenvironment, we evaluated specific immunosuppressive networks. Interactomic analysis revealed that malignant TSKs and stromal mCAFs participated in broader predicted antigen-presentation-related communication networks involving CD8^+^ Tem cells through MHC-I signaling and Tregs through MHC-II signaling (**Supplementary Figures 4E, F**). However, alongside these antigen-presentation-related interactions, we observed a concurrent network of coinhibitory signaling. Specifically, bubble plot analysis identified a prominent NECTIN2–TIGIT suppressive axis mediating contact-dependent inhibitory crosstalk from TSKs, mCAFs, and Angio-TAMs toward Tregs (35). Furthermore, these checkpoint interactions were accompanied by SPP1–CD44 signaling from Angio-TAMs toward CD4^+^ naive T cells, CD8^+^ Tem cells, and Tregs, together with SPP1–(ITGA4^+^ITGB1) signaling toward CD8^+^ Tem cells (**Figure 4G**) (36). Collectively, these predicted signaling profiles suggest that TSKs, mCAFs, and Angio-TAMs may cooperatively shape an immunosuppressive microenvironment through complementary NECTIN2–TIGIT checkpoint signaling and SPP1-mediated interactions with T-cell subsets.

To further assess the spatial organization of key cellular populations implicated in these signaling networks in situ, we performed multiplex immunofluorescence analysis on matched cSCC tissues, with corresponding H&E-stained sections provided for histological reference (**Supplementary Figures 4G, H**). Spatial analysis showed that CD163^+^SPP1^+^ Angio-TAMs were more abundant in tumor tissues than in matched paratumoral regions (**Figures 4H, I**). Notably, a subset of these Angio-TAMs was observed adjacent to α-SMA^+^ CAF-rich stromal areas near the tumor invasive front (**Figure 4H**). These observations provide *in situ* support for the spatial association between Angio-TAMs and CAF-enriched stromal niches in cSCC, providing spatial context for the cellular networks identified by single-cell transcriptomic analysis.

### 3.5 NR1H3 characterizes Angio-TAMs and is associated with poor survival in SCC

**Figure 5:**
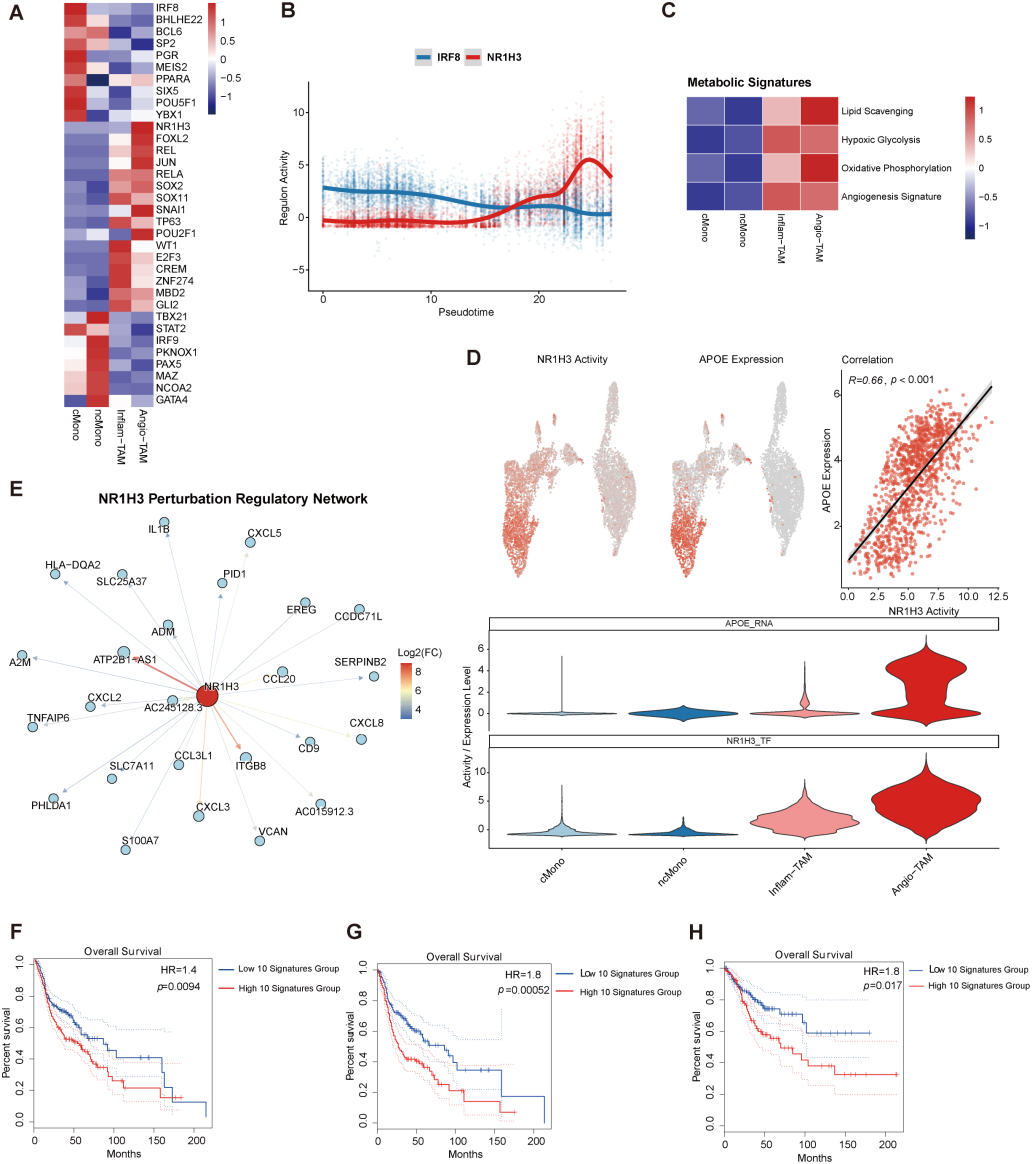
NR1H3 characterizes the lipid-associated Angio-TAM state and is associated with poor survival across SCC cohorts. **(A)** Heatmap depicting transcription factor activity scores across the monocytic and macrophage subpopulations inferred using decoupleR with the DoRothEA prior knowledge network. Red indicates high activity, while blue indicates low activity. **(B)** Line plot illustrating the dynamic shifts in regulon activity for *IRF8* (blue) and *NR1H3* (red) along the inferred pseudotime developmental trajectory from monocytes to Angio-TAMs. **(C)** Heatmap evaluating custom metabolic signatures, showing elevated lipid-scavenging and hypoxic-glycolysis signatures in the Angio-TAM subset. **(D)** UMAP feature plots (top left and center) and a Pearson correlation scatter plot (top right) demonstrating the strong positive correlation (R = 0.66, p < 0.001 between *NR1H3* regulon activity and *APOE* gene expression. Stacked violin plots (bottom) further illustrate the preferential enrichment of NR1H3 activity and APOE expression in Angio-TAMs. **(E)** Network graph illustrating the downstream targets predicted to be affected by *in silico* virtual knockout of *NR1H3*. Node size and color represent the predicted perturbation magnitude, highlighting the potential regulatory effects of NR1H3 on key inflammatory and matrix-remodeling effectors. **(F–H)** Kaplan-Meier overall survival curves for patients stratified into high- and low-expression groups based on the median score of the *NR1H3*-associated 10-gene signature across three independent TCGA SCC cohorts: HNSC **(F)**, LUSC **(G)**, and CESC **(H)**. Statistical significance was determined by the log-rank test.

To elucidate the regulatory mechanisms orchestrating the polarization of Angio-TAMs, we inferred single-cell transcription factor activities utilizing the decoupleR algorithm coupled with the DoRothEA prior knowledge network. We identified dynamic shifts in transcription factor activities driving the monocytic-to-TAM transition. While the activity of early monocytic lineage regulators, such as *IRF8*, was progressively repressed, the nuclear receptor *NR1H3* markedly increased during the terminal differentiation of Angio-TAMs (**Figures 5A, B**). Cross-lineage comparison further showed that NR1H3 was preferentially enriched in Angio-TAMs at both the transcript and regulatory activity levels (**Supplementary Figures 5A, B**). Given the established role of NR1H3 in lipid homeostasis (37), we evaluated metabolic signatures and confirmed that Angio-TAMs displayed elevated lipid-scavenging and hypoxic-glycolysis signatures (**Figure 5C**), with the lipid-related metabolic profile further supported by complementary GSVA analysis (**Supplementary Figure 5C**). Furthermore, expression profiling and correlation analyses revealed that APOE, a core lipid transporter and known mediator of tumor immunosuppression (38), was not only specifically enriched in the Angio-TAM subset but also exhibited a concordant single-cell distribution and a strong positive correlation (R = 0.66, *p* < 0.001) with *NR1H3* activity, suggesting a potential regulatory relationship (**Figure 5D**).

To evaluate the potential regulatory impact of *NR1H3* on the Angio-TAM functional state, we performed *in silico* gene perturbation. Virtual knockout of *NR1H3* was predicted to substantially perturb the downstream regulatory network (**Figure 5E**). Notably, NR1H3 perturbation strongly affected critical structural and inflammatory effectors, including the core extracellular matrix component *VCAN* (39) and the immunoregulatory integrin *ITGB8* (40) and a distinct array of chemokines (*CXCL2*, *CXCL3*, *CXCL5*, *CXCL8*, *CCL20*) (**Supplementary Figure 5D**). GO and KEGG pathway analyses showed that these perturbed targets were significantly enriched in processes related to myeloid leukocyte chemotaxis, chemokine-mediated signaling, and cytokine-cytokine receptor interactions (**Supplementary Figures 5E, F**). Collectively, these data suggest that NR1H3 may extend beyond lipid homeostasis and function as a central transcriptional node associated with the maintenance of the characteristic chemotactic and matrix-remodeling networks of the Angio-TAM subpopulation.

To experimentally support the predicted NR1H3-associated Angio-TAM program, we performed *in vitro* validation using A431-conditioned medium. A431-conditioned medium modestly increased NR1H3 expression and markedly induced APOE expression in THP-1-derived macrophage-like cells, whereas siRNA-mediated NR1H3 knockdown attenuated APOE upregulation (**Supplementary Figure 5G**). In parallel, representative invasion assays suggested that conditioned supernatants from A431-conditioned medium-educated macrophage-like cells enhanced A431 cell invasion, while NR1H3 knockdown reduced this pro-invasive effect (**Supplementary Figure 5H**). These findings support a role for NR1H3 in maintaining an APOE-associated, pro-invasive macrophage program induced by cSCC-derived signals.

Recognizing the potential importance of this NR1H3-driven program in the cSCC microenvironment, we sought to evaluate its broader clinical significance. We derived a 10-gene signature representing the NR1H3-driven Angio-TAM program and evaluated its prognostic value across independent TCGA cohorts of squamous cell carcinomas. Strikingly, Kaplan-Meier survival analysis revealed that patients with high expression of the *NR1H3*-driven Angio-TAM signature exhibited significantly worse overall survival in head and neck squamous cell carcinoma (HNSC) (*p* = 0.0094, HR = 1.4), lung squamous cell carcinoma (LUSC) (*p* = 0.00052, HR = 1.8), and cervical squamous cell carcinoma (CESC) (*p* = 0.017, HR = 1.8) (**Figures 5F–H**). Collectively, these findings support the broader prognostic relevance of the NR1H3-driven Angio-TAM signature across multiple SCC cohorts.

### 3.6 Independent external validation confirms the Angio-TAM phenotype and NR1H3-associated molecular program

**Figure 6:**
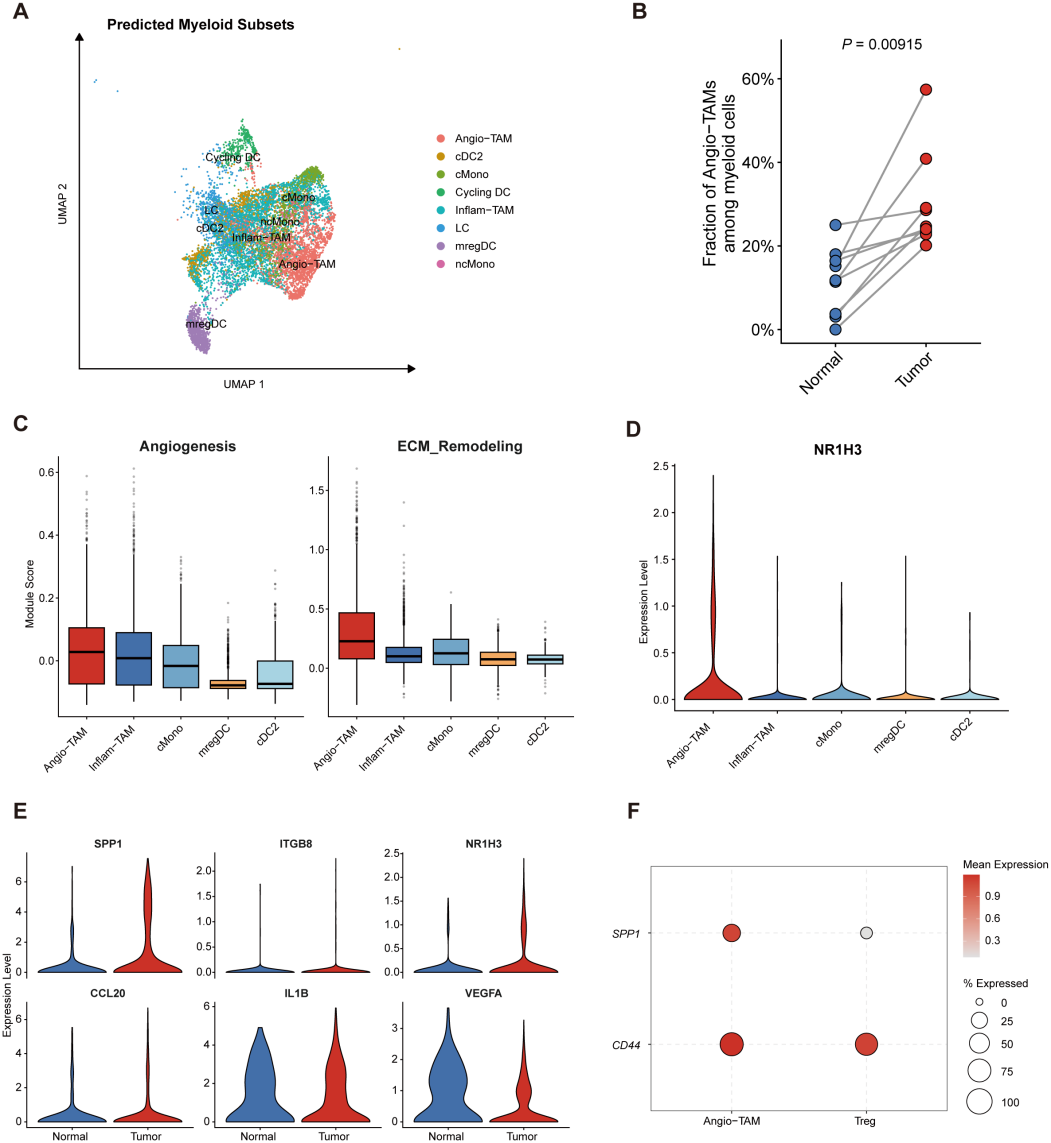
Independent external validation of the Angio-TAM phenotype and the NR1H3-associated molecular program. **(A)** UMAP plot depicting the predicted myeloid subsets in an independent cSCC validation cohort, identified via Canonical Correlation Analysis (CCA)-based label transfer and reference mapping. **(B)** Paired sample-level comparison of Angio-TAM fractions among myeloid cells between matched normal skin and cSCC tumor tissues in the validation cohort. Lines connect paired samples from the same patient. Statistical significance was assessed using a paired Wilcoxon signed-rank test. **(C)** Box plots evaluating the angiogenesis (left) and ECM-remodeling (right) module scores across the predicted myeloid subpopulations, showing that Angio-TAMs exhibited the highest scores for both transcriptional programs. **(D)** Violin plot demonstrating the preferential expression of NR1H3 in the validated Angio-TAM subset. **(E)** Split violin plots comparing the expression levels of selected Angio-TAM-associated genes (*SPP1*, *ITGB8*, *NR1H3*, *CCL20*, *IL1B*, and *VEGFA*) between normal and tumor tissues. **(F)** Dot plot showing expression patterns supporting a potential SPP1–CD44 signaling relationship between Angio-TAMs and Tregs. SPP1 was predominantly expressed in Angio-TAMs, whereas CD44 was highly expressed in Tregs and was also expressed in Angio-TAMs. The dot size represents the percentage of cells expressing the respective gene, and the color intensity indicates the mean expression level.

To ensure the generalizability and robustness of our findings, we sought to validate the Angio-TAM phenotype and its associated transcriptional features in an independent single-cell cSCC cohort (GEO: GSE144240) (12). We employed Canonical Correlation Analysis (CCA)-based reference mapping to project our finely resolved myeloid annotations onto this query validation dataset (**Figure 6A**). This label transfer confirmed the presence of all major myeloid populations, including the highly specialized Angio-TAM subset. Ultimately, this external validation confirms that the Angio-TAM population is a biologically conserved entity across diverse patient cohorts, rather than an artifact of a specific dataset.

Consistent with the discovery cohort, paired sample-level analysis in the validation cohort showed that the fraction of Angio-TAMs among myeloid cells was significantly increased in tumor tissues compared with matched normal skin samples (*p* = 0.00915) (**Figure 6B**). Furthermore, functional module scoring demonstrated that these Angio-TAMs exhibited the highest activity scores for angiogenesis and ECM remodeling among all evaluated myeloid subsets (**Figure 6C**). These results corroborate our initial findings, supporting the strong association of the Angio-TAM subpopulation with pro-tumorigenic and microenvironment-remodeling transcriptional programs.

Next, we evaluated the molecular features of this validated subpopulation. Consistent with our initial dataset, *NR1H3* expression remained preferentially enriched in the Angio-TAM subset (**Figure 6D**). Furthermore, both *NR1H3* and the core effector *SPP1* were upregulated in tumor lesions compared to normal tissues (**Figure 6E**). Conversely, classical inflammatory and angiogenic factors, including *IL1B*, *CCL20*, and *VEGFA*, exhibited comparable or even higher baseline expression in the normal skin microenvironment. Finally, expression profiling supported a potential SPP1-CD44 communication axis: SPP1 was highly expressed by Angio-TAMs, while its receptor CD44 was highly expressed on both Angio-TAMs and Tregs (**Figure 6F**). Collectively, this external validation supports the reproducibility of an NR1H3-associated Angio-TAM phenotype and a potential SPP1–CD44 signaling relationship with Tregs in an independent cSCC cohort.

## 4 Discussion

The tumor microenvironment is increasingly recognized as a critical determinant of tumor progression, immune regulation, and therapeutic response in solid malignancies (41, 42). In this study, we constructed a comprehensive single-cell landscape of the cSCC microenvironment using matched tumor tissues, adjacent normal skin, and peripheral blood samples. By integrating lineage trajectory analysis, transcriptional regulatory inference, and intercellular communication modeling, we delineated the multicellular architecture of cSCC and identified coordinated interactions among epithelial, stromal, and immune compartments. These analyses revealed a TAM state characterized by angiogenic and matrix-remodeling programs, and highlighted the nuclear receptor NR1H3 as a core transcriptional regulator associated with this macrophage phenotype.

Macrophages represent one of the most abundant immune populations in the tumor microenvironment and exhibit remarkable functional plasticity (43, 44). Increasing evidence indicates that TAMs can arise from recruited circulating monocytes and subsequently acquire specialized phenotypes shaped by local microenvironmental signals (9, 45, 46). Consistent with this paradigm, our trajectory analysis reconstructed a differentiation continuum extending from circulating monocytes toward distinct macrophage states within tumor tissues. Notably, one macrophage population displayed transcriptional programs enriched for angiogenesis, extracellular matrix remodeling, and chemotactic signaling, suggesting the emergence of a tumor-adapted macrophage state specialized for shaping the vascular and stromal landscape of the tumor niche (8). This transcriptional profile is consistent with known pro-tumor functions of TAMs, particularly in promoting angiogenesis, matrix remodeling, and cancer dissemination (47). In addition, recent studies have linked SPP1-associated macrophage states to tissue remodeling and immune suppression, supporting the notion that Angio-TAMs may contribute to both stromal conditioning and immune evasion in cSCC (48). More broadly, functionally similar pro-angiogenic macrophage subsets have been identified in several cancers, including hepatocellular carcinoma and HNSC (31, 49, 50), suggesting that specialized angiogenic macrophages may represent a conserved feature of tumor-associated myeloid ecosystems.

Beyond immune cells, stromal remodeling represents another fundamental component of tumor ecosystem evolution. CAFs have emerged as highly heterogeneous populations that regulate tumor progression through matrix deposition, inflammatory signaling, and modulation of immune cell recruitment (51, 52). Previous studies have demonstrated that fibroblast populations can differentiate into distinct functional states, including inflammatory CAFs and matrix-producing CAFs, which collectively shape tumor architecture and mechanical properties (53, 54). In our dataset, trajectory inference revealed a differentiation continuum linking steady-state fibroblasts to specialized CAF states with distinct inflammatory and matrix-remodeling signatures. Notably, matrix-associated CAFs were preferentially enriched in paratumoral tissues, suggesting that stromal remodeling may be particularly prominent within the tumor-adjacent stromal compartment of cSCC. Such stromal remodeling has been proposed to regulate tumor invasion by altering extracellular matrix composition and mechanical constraints within the tumor microenvironment (55, 56).

Tumor progression is increasingly understood as a multicellular process governed by extensive intercellular communication networks. Ligand–receptor signaling between tumor cells, stromal populations, and immune infiltrates coordinates key biological processes including immune suppression, angiogenesis, and extracellular matrix remodeling (57, 58). Through systematic communication analysis, we identified dense signaling networks linking macrophages, fibroblasts, and malignant keratinocytes. These interactions were enriched for pathways involved in extracellular matrix organization, cytokine signaling, and immune regulatory processes. In particular, our communication models suggest that Angio-TAMs, together with CAF subsets and malignant keratinocytes, may participate in immunoregulatory circuits involving checkpoint-related and SPP1-mediated signaling, which have been implicated in tumor immune evasion (59, 60). The presence of coordinated stromal and immune signaling networks in our dataset further supports the concept that tumor ecosystems are maintained through dynamic multicellular interactions rather than the activity of individual cell types (61).

A central finding of our study is the identification of a transcriptional regulatory program associated with the angiogenic macrophage state. Transcription factor activity inference highlighted NR1H3 as a candidate regulator with preferential activity in this macrophage population. *NR1H3* encodes liver X receptor α, a lipid-sensing nuclear receptor that integrates metabolic signals with transcriptional regulation in macrophages (62, 63). NR1H3 signaling has been shown to regulate cholesterol homeostasis, inflammatory responses, and macrophage survival, thereby linking metabolic state to immune function (64, 65). Emerging evidence suggests that metabolic reprogramming plays a central role in shaping macrophage polarization within tumors (66–68). Our findings extend these observations by suggesting that NR1H3 may coordinate transcriptional programs associated with angiogenesis, chemokine signaling, and extracellular matrix remodeling in TAMs. Consistent with this inference, our cellular validation showed that NR1H3 knockdown attenuated cSCC-conditioned medium-induced APOE upregulation and reduced the pro-invasive effect of macrophage-conditioned supernatants, providing experimental support for a functional role of NR1H3 within this macrophage program.

Importantly, the regulatory axis identified in our study may have broader relevance beyond cSCC. Squamous cell carcinomas arising in different organs share common molecular and immunological features, including strong inflammatory signaling and complex stromal remodeling (69, 70). Consistent with this notion, the *NR1H3*-associated transcriptional signature identified in our analysis correlated with poor clinical outcomes across multiple squamous cell carcinoma cohorts, including head and neck, lung, and cervical cancers. The biological plausibility of this clinical association is further supported by several representative components within the 10-gene signature. For instance, SPP1 has been associated with macrophage-driven progression in HNSC and with tumor–macrophage crosstalk in esophageal squamous cell carcinoma (50, 71), whereas CXCL8-associated stromal and inflammatory programs have been implicated in high-risk cSCC and invasive progression in squamous tumors (14, 72, 73). Likewise, EREG has been associated with oncogenic transformation, treatment resistance, and worse outcomes in squamous carcinomas (74, 75). Together, these findings support the biological relevance of the NR1H3-associated 10-gene signature beyond cSCC and suggest that macrophage regulatory programs identified in cSCC may reflect shared biological features across squamous malignancies. In light of the growing interest in targeting tumor-associated macrophages as a therapeutic strategy, transcriptional regulators that coordinate macrophage polarization may represent candidate targets for microenvironment-directed therapies (76).

Several limitations should be considered when interpreting these findings. First, although single-cell transcriptomics provides a powerful framework for resolving cellular heterogeneity, transcriptional data alone cannot fully capture spatial organization or functional interactions within tumor tissues. Although our multiplex imaging provided initial spatial validation, integration with higher-resolution spatial transcriptomics will be necessary to further define the spatial architecture of macrophage–fibroblast–epithelial interactions in the cSCC microenvironment. Second, although our computational analyses and cellular validation suggest a regulatory role for NR1H3 in macrophage polarization, further in vivo and mechanistic studies will be required to establish its causal function, define its direct downstream targets, and determine how metabolic signaling pathways influence macrophage behavior during tumor progression.

In summary, our study provides a comprehensive single-cell characterization of the cSCC microenvironment and highlights the coordinated multicellular interactions that shape tumor progression. By identifying a macrophage program associated with angiogenesis and a potential regulatory role for NR1H3, these findings expand our understanding of immune and stromal dynamics in squamous carcinomas and provide a framework for future studies exploring microenvironment-targeted therapeutic strategies.

## Data Availability

The publicly available datasets analyzed for this study can be found in the Gene Expression Omnibus (GEO) repository GSE144240; https://www.ncbi.nlm.nih.gov/geo/query/acc.cgi?acc=GSE144240. The original single-cell sequencing datasets generated for this study have been deposited in the Genome Sequence Archive (Genomics, Proteomics & Bioinformatics 2025) in National Genomics Data Center (Nucleic Acids Res 2025), China National Center for Bioinformation / Beijing Institute of Genomics, Chinese Academy of Sciences (GSA-Human: HRA017510) that are publicly accessible at https://ngdc.cncb.ac.cn/gsa-human.
