# Supplementary material for: Single-cell transcriptomic analysis of cutaneous squamous cell carcinoma identifies an NR1H3-associated angiogenic macrophage state
Source: Front Immunol. 2026 Jul 30;17:1837479. doi: 10.3389/fimmu.2026.1837479 (PMC13479357; doi:10.3389/fimmu.2026.1837479)
Supplement: Supplementary file 1 [file DataSheet1.pdf]

Supplementary Material

1 Supplementary Figures and Tables

1.1 Supplementary Figures

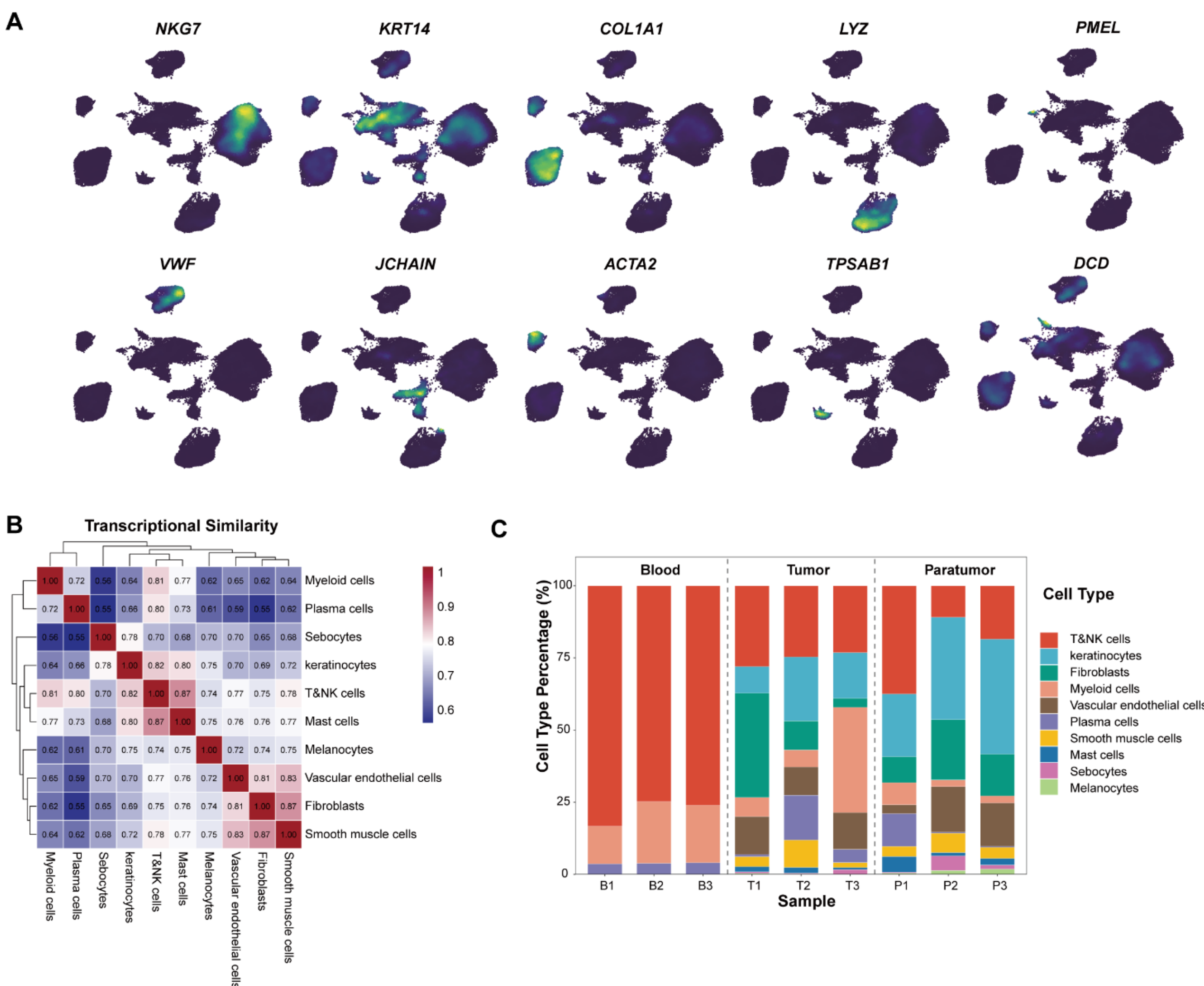

Supplementary Figure 1 Validation of cell type annotations and sample-level distributions in the cSCC landscape.

(A) Feature plots demonstrating the expression levels and distribution of representative lineage marker genes (*NKG7, KRT14, COL1A1, LYZ, PMEL, VWF, JCHAIN, ACTA2, TPSAB1*, and *DCD*) projected onto the UMAP embedding. The brighter, luminous yellow-green colors indicate higher

expression levels against the dark background. **(B)** Correlation heatmap displaying the global transcriptional similarity among the identified major cell lineages. **(C)** Stacked bar plot detailing the relative proportion of major cell types within each individual sample, categorized by tissue origin (Blood: B1-B3; Tumor: T1-T3; Paratumor: P1-P3).

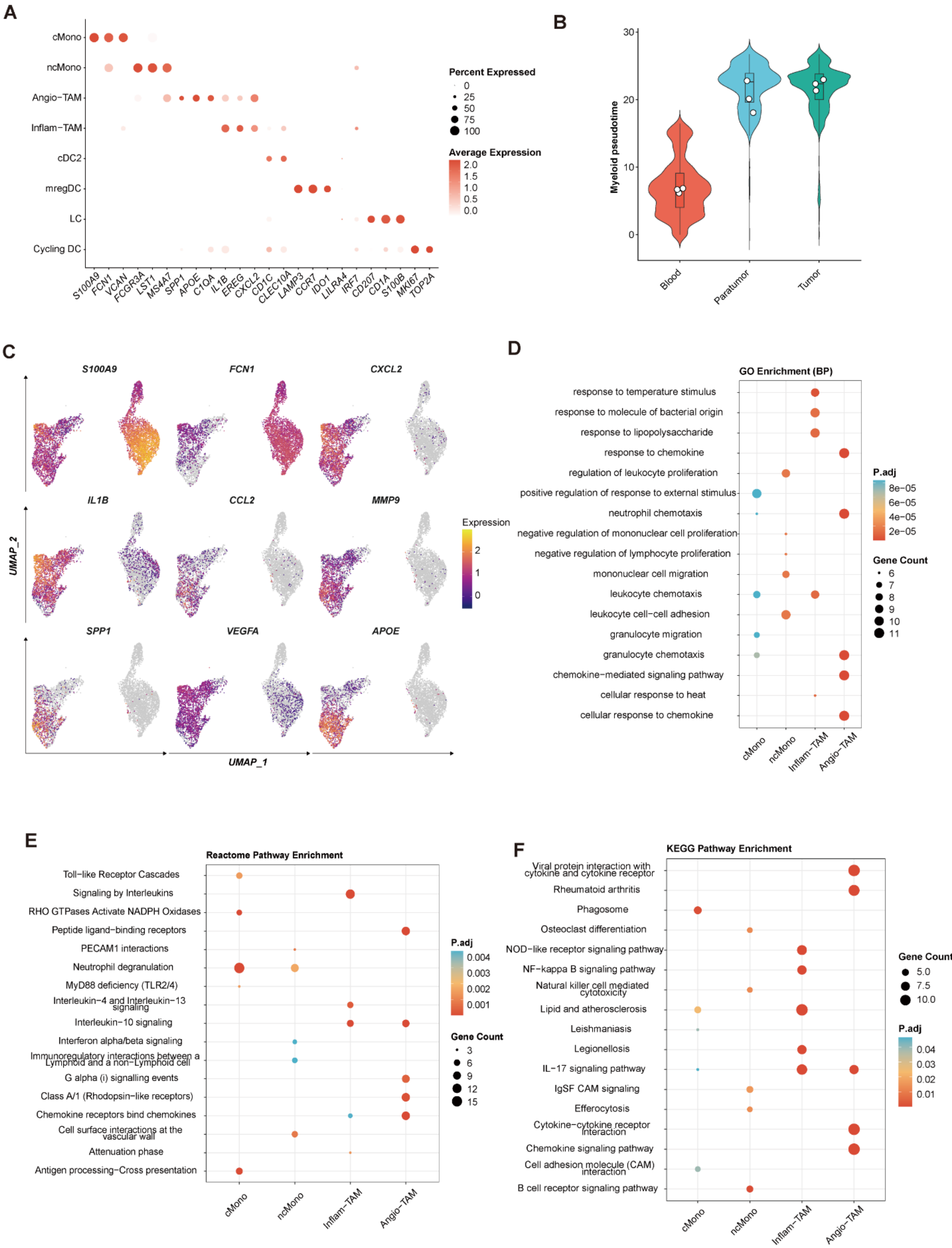
A
cMono
ncMono
Angio−TAM
Inflam−TAM
cDC2
mregDC
LC
Cycling DC
S100A9
FCN1
VCAN
FCGR3A
LST1
MS4A7
SPP1
APOE
C1QA
IL1B
EREG
CXCL2
CD1C
CLEC10A
LAMP3
CCR7
IDO1
LILRA4
IRF7
CD207
CD1A
S100B
MKI67
TOP2A
Percent Expressed
0
25
50
75
100
Average Expression
2.0
1.5
1.0
0.5
0.0
B
Myeloid pseudotime
30
20
10
0
Blood
Paratumor
Tumor
C
S100A9
FCN1
CXCL2
IL1B
CCL2
MMP9
SPP1
VEGFA
APOE
UMAP_2
UMAP_1
Expression
2
1
0
D
GO Enrichment (BP)
response to temperature stimulus
response to molecule of bacterial origin
response to lipopolysaccharide
response to chemokine
regulation of leukocyte proliferation
positive regulation of response to external stimulus
neutrophil chemotaxis
negative regulation of mononuclear cell proliferation
negative regulation of lymphocyte proliferation
mononuclear cell migration
leukocyte chemotaxis
leukocyte cell−cell adhesion
granulocyte migration
granulocyte chemotaxis
chemokine−mediated signaling pathway
cellular response to heat
cellular response to chemokine
cMono
ncMono
Inflam−TAM
Angio−TAM
P.adj
8e−05
6e−05
4e−05
2e−05
Gene Count
6
7
8
9
10
11
E
Reactome Pathway Enrichment
Toll−like Receptor Cascades
Signaling by Interleukins
RHO GTPases Activate NADPH Oxidases
Peptide ligand−binding receptors
PECAM1 interactions
Neutrophil degranulation
MyD88 deficiency (TLR2/4)
Interleukin−4 and Interleukin−13 signaling
Interleukin−10 signaling
Interferon alpha/beta signaling
Immunoregulatory interactions between a Lymphoid and a non−Lymphoid cell
G alpha (i) signalling events
Class A/1 (Rhodopsin−like receptors)
Chemokine receptors bind chemokines
Cell surface interactions at the vascular wall
Attenuation phase
Antigen processing−Cross presentation
cMono
ncMono
Inflam−TAM
Angio−TAM
P.adj
0.004
0.003
0.002
0.001
Gene Count
3
6
9
12
15
F
KEGG Pathway Enrichment
Viral protein interaction with cytokine and cytokine receptor
Rheumatoid arthritis
Phagosome
Osteoclast differentiation
NOD−like receptor signaling pathway
NF−kappa B signaling pathway
Natural killer cell mediated cytotoxicity
Lipid and atherosclerosis
Leishmaniasis
Legionellosis
IL−17 signaling pathway
IgSF CAM signaling
Efferocytosis
Cytokine−cytokine receptor interaction
Chemokine signaling pathway
Cell adhesion molecule (CAM) interaction
B cell receptor signaling pathway
cMono
ncMono
Inflam−TAM
Angio−TAM
Gene Count
5.0
7.5
10.0
P.adj
0.04
0.03
0.02
0.01

Supplementary Figure 2 Transcriptional signatures and functional pathway enrichment of the myeloid compartment.

(A) Dot plot detailing the expression of canonical and specific marker genes across all eight identified myeloid subsets. The dot size represents the percentage of cells expressing the respective gene, and the color intensity indicates the average expression level. **(B)** Violin plots showing myeloid pseudotime values across peripheral blood, paratumor, and tumor compartments. White dots indicate sample-level median pseudotime values. **(C)** Feature plots demonstrating the expression levels and distribution of representative genes driving the monocytic (*S100A9*, *FCN1*), inflammatory (*CXCL2*, *IL1B*, *CCL2*), and pro-angiogenic/remodeling (*MMP9*, *SPP1*, *VEGFA*, *APOE*) phenotypes projected onto the UMAP embedding. Darker colors (yellow/red) indicate higher expression levels. **(D–F)** Dot plots illustrating the top enriched terms from Gene Ontology (GO) biological process **(D)**, Reactome pathway **(E)**, and KEGG pathway **(F)** enrichment analyses for cMono, ncMono, Inflam-TAM, and Angio-TAM subsets. The dot size represents the number of genes involved in the pathway, and the color gradient indicates the statistical significance (*p.adj* value).

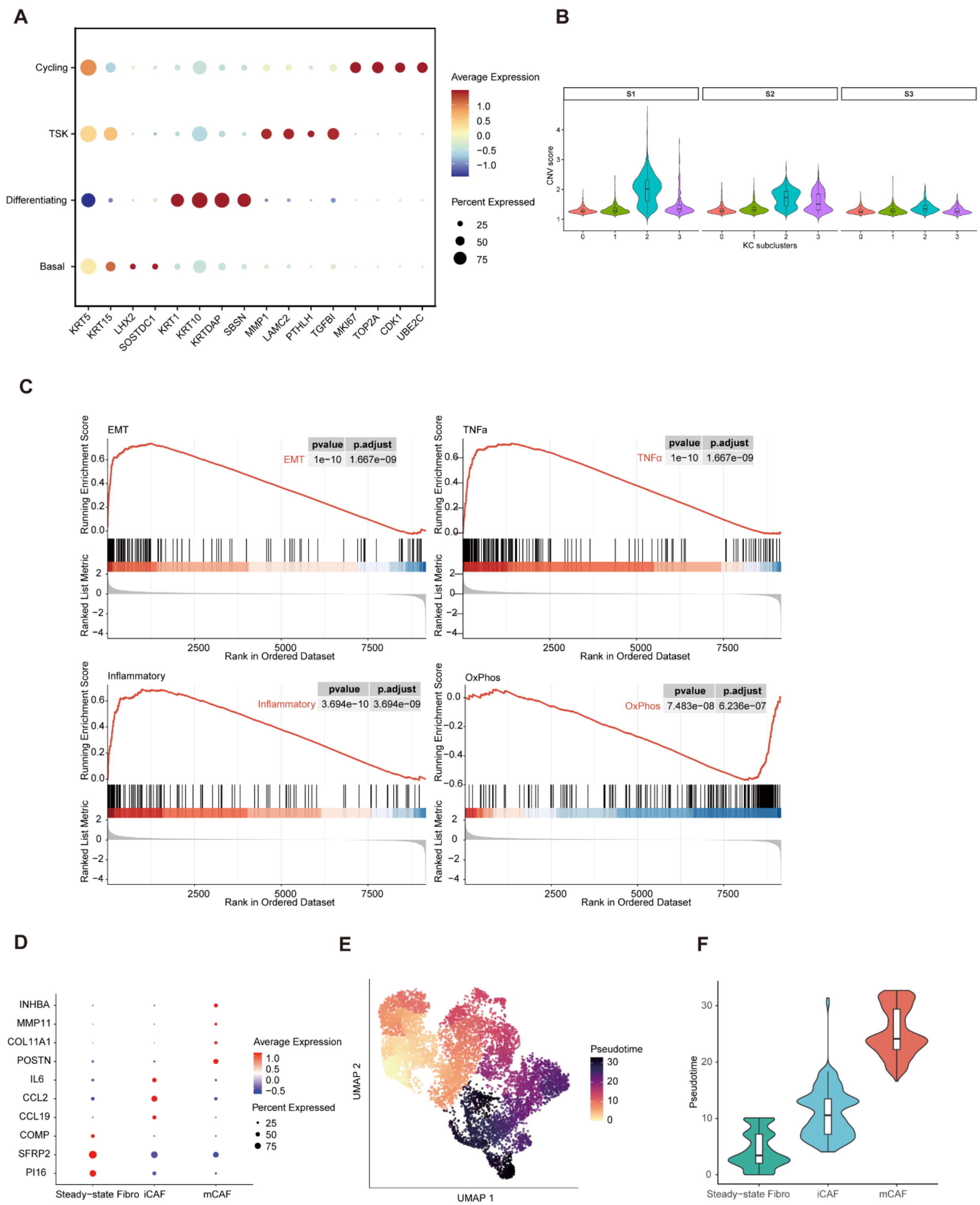
A
Cycling
TSK
Differentiating
Basal
KRT5
KRT15
LHX2
SOSTDC1
KRT1
KRT10
KRTDAP
SBSN
MMP1
LAMC2
PTHLH
TGFBI
MKI67
TOP2A
CDK1
UBE2C
Average Expression
1.0
0.5
0.0
−0.5
−1.0
Percent Expressed
25
50
75
B
S1
S2
S3
CNV score
KC subclusters
C
EMT
TNFa
Inflammatory
OxPhos
pvalue
p.adjust
EMT 1e−10 1.667e−09
TNFα 1e−10 1.667e−09
Inflammatory 3.694e−10 3.694e−09
OxPhos 7.483e−08 6.236e−07
Running Enrichment Score
Ranked List Metric
Rank in Ordered Dataset
2500
5000
7500
D
INHBA
MMP11
COL11A1
POSTN
IL6
CCL2
CCL19
COMP
SFRP2
PI16
Steady−state Fibro
iCAF
mCAF
Average Expression
1.0
0.5
0.0
−0.5
Percent Expressed
25
50
75
E
UMAP 1
UMAP 2
Pseudotime
30
20
10
0
F
Pseudotime
Steady−state Fibro
iCAF
mCAF

Supplementary Figure 3 Molecular profiling and pathway enrichment of keratinocyte and fibroblast subpopulations.

(A) Dot plot detailing the expression of canonical and state-specific marker genes across the four keratinocyte subpopulations. The dot size represents the percentage of cells expressing the respective gene, and the color intensity indicates the average expression level. **(B)** Sample-stratified violin plots showing inferred CNV scores of keratinocyte subclusters within each individual cSCC sample. **(C)** GSEA plots demonstrating the enrichment of epithelial-mesenchymal transition (EMT), TNF-α, and inflammatory signatures, alongside the concurrent downregulation of oxidative phosphorylation (OxPhos) in the malignant TSK subset. **(D)** Dot plot illustrating the expression profiles of key marker genes used to define the Steady-state Fibroblast, iCAF, and mCAF populations. **(E)** UMAP plot of the fibroblast compartment colored by the inferred pseudotime values, with lighter colors representing earlier developmental stages and darker colors indicating terminal differentiation. **(F)** Violin plot quantifying the distribution of pseudotime values across the three fibroblast subsets, confirming mCAFs as the most advanced differentiated state within the trajectory.

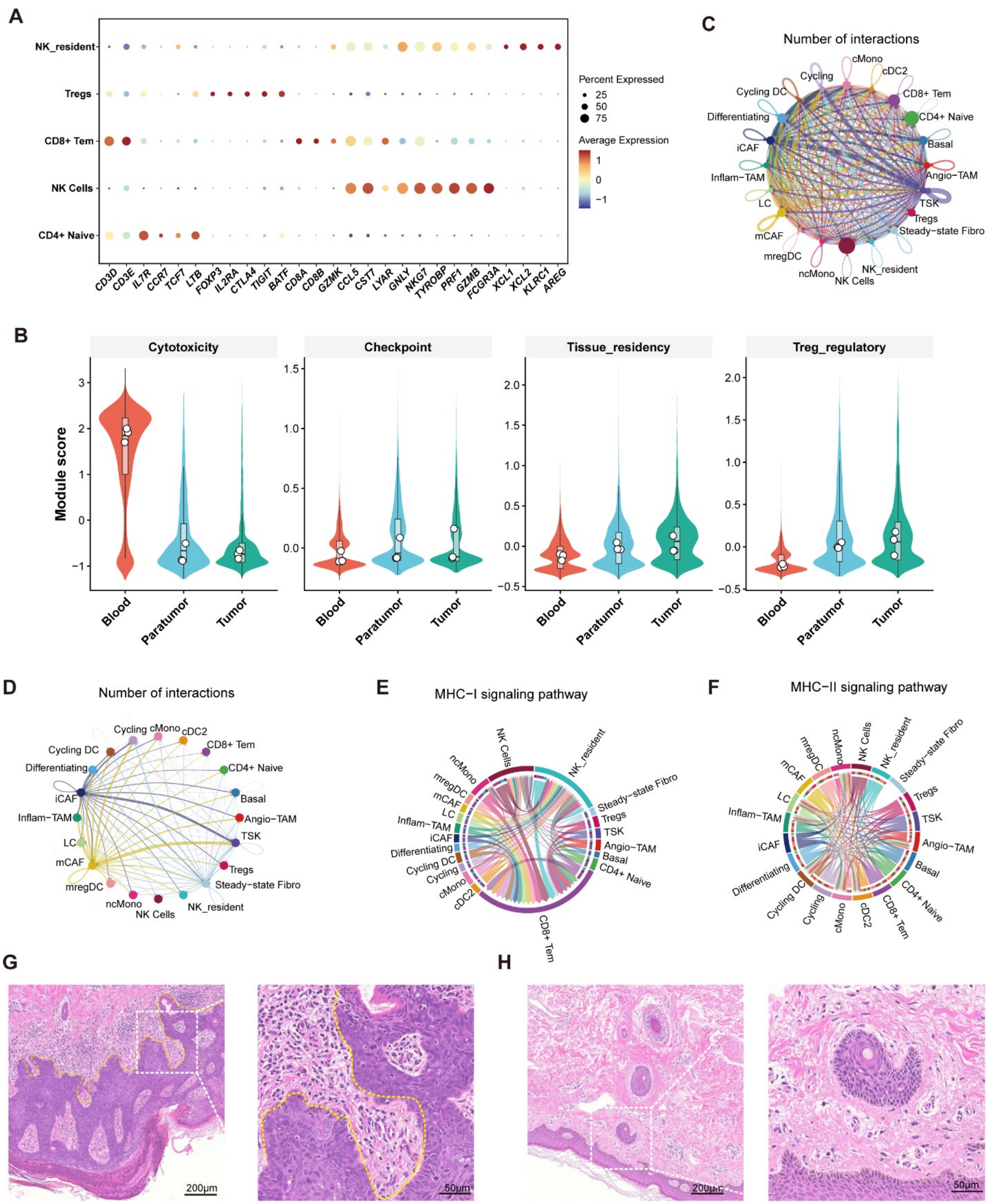

Supplementary Figure 4 Molecular validation of immune subclusters and extended intercellular signaling networks.

(A) Dot plot detailing the expression of canonical and specific marker genes utilized to annotate the five immune subpopulations. The dot size represents the percentage of cells expressing the respective

gene, and the color intensity indicates the average expression level. **(B)** Violin plots showing cytotoxicity, checkpoint-related, tissue-residency, and Treg/immunoregulatory module scores in T&NK cells across peripheral blood, paratumor, and tumor compartments. White dots indicate sample-level median scores. **(C)** Global network plot indicating the total number of inferred interactions among all identified cell types. **(D)** Network plot displaying the total number of inferred interactions within the extracellular matrix (ECM) signaling pathway. **(E, F)** Chord diagrams illustrating predicted antigen-presentation-related communication networks mediated by the MHC-I **(E)** and MHC-II **(F)** signaling pathways across epithelial, stromal, and immune compartments. **(G)** Representative H&E-stained images of cSCC tumor tissue at low magnification (10×, left) and high magnification (40×, right). The yellow dashed lines indicate the invasive front, defined as the interface between invading tumor nests and the surrounding stromal compartment. Scale bars: 200 µm (left) and 50 µm (right). **(H)** Representative H&E-stained images of matched paratumor tissue at low magnification (10×, left) and high magnification (40×, right). Scale bars: 200 µm (left) and 50 µm (right).

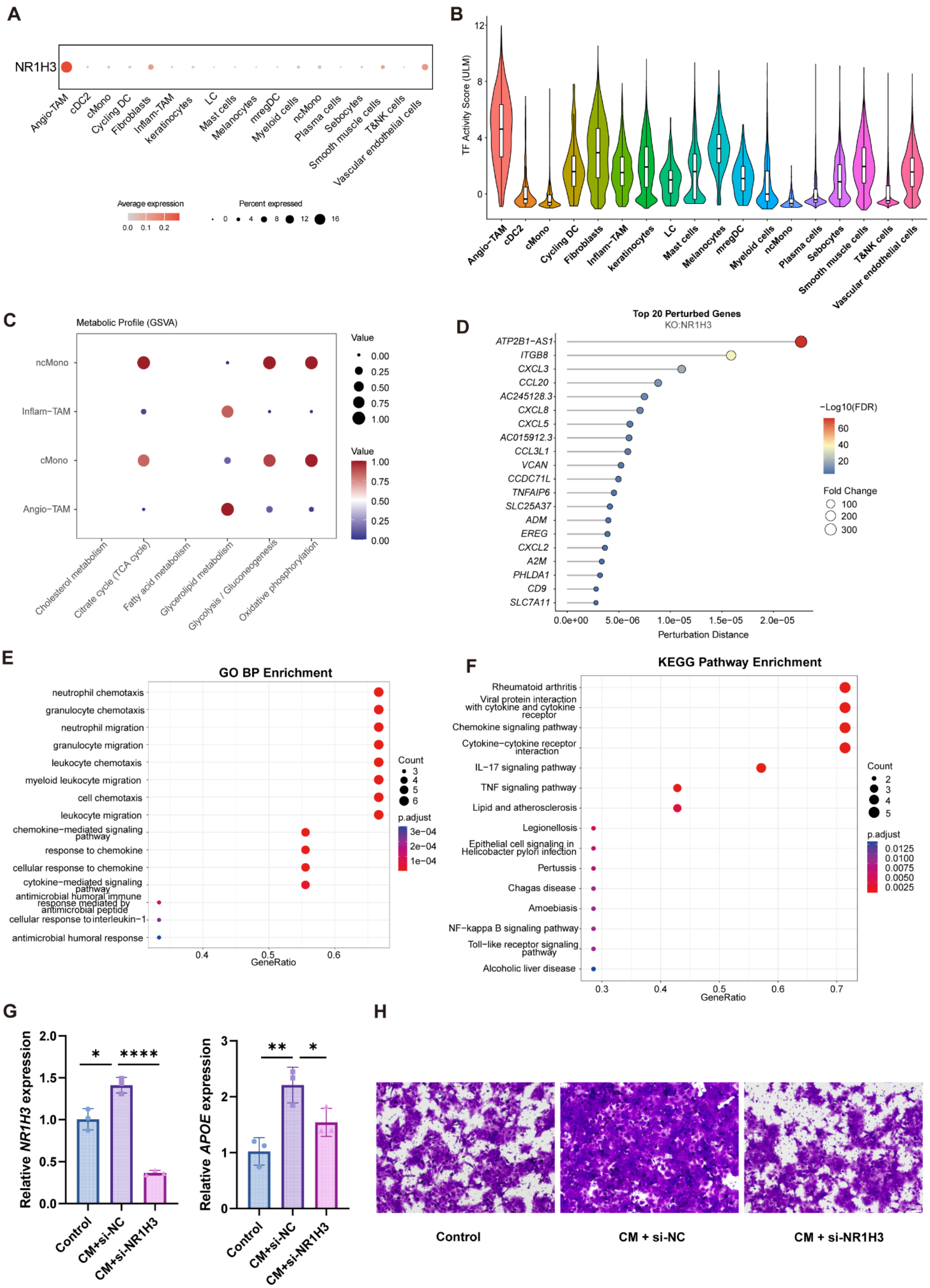
A
NR1H3
Angio-TAM
cDC2
cMono
Cycling DC
Fibroblasts
Inflam-TAM
keratinocytes
LC
Mast cells
Melanocytes
mregDC
Myeloid cells
ncMono
Plasma cells
Sebocytes
Smooth muscle cells
T&NK cells
Vascular endothelial cells
Average expression
0.0 0.1 0.2
Percent expressed
0 4 8 12 16
B
TF Activity Score (ULM)
C
Metabolic Profile (GSVA)
Value
Cholesterol metabolism
Citrate cycle (TCA cycle)
Fatty acid metabolism
Glycerolipid metabolism
Glycolysis / Gluconeogenesis
Oxidative phosphorylation
D
Top 20 Perturbed Genes
KO:NR1H3
ATP2B1−AS1
ITGB8
CXCL3
CCL20
AC245128.3
CXCL8
CXCL5
AC015912.3
CCL3L1
VCAN
CCDC71L
TNFAIP6
SLC25A37
ADM
EREG
CXCL2
A2M
PHLDA1
CD9
SLC7A11
−Log10(FDR)
Fold Change
Perturbation Distance
E
GO BP Enrichment
neutrophil chemotaxis
granulocyte chemotaxis
neutrophil migration
granulocyte migration
leukocyte chemotaxis
myeloid leukocyte migration
cell chemotaxis
leukocyte migration
chemokine−mediated signaling pathway
response to chemokine
cellular response to chemokine
cytokine−mediated signaling pathway
antimicrobial humoral immune response mediated by antimicrobial peptide
cellular response to interleukin−1
antimicrobial humoral response
Count
p.adjust
GeneRatio
F
KEGG Pathway Enrichment
Rheumatoid arthritis
Viral protein interaction with cytokine and cytokine receptor
Chemokine signaling pathway
Cytokine−cytokine receptor interaction
IL−17 signaling pathway
TNF signaling pathway
Lipid and atherosclerosis
Legionellosis
Epithelial cell signaling in Helicobacter pylori infection
Pertussis
Chagas disease
Amoebiasis
NF−kappa B signaling pathway
Toll−like receptor signaling pathway
Alcoholic liver disease
Count
p.adjust
GeneRatio
G
Relative NR1H3 expression
Relative APOE expression
Control
CM+si-NC
CM+si-NR1H3
H
Control
CM + si-NC
CM + si-NR1H3

Supplementary Figure 5 NR1H3 expression, regulatory activity, and downstream perturbation analysis.

(A) Dot plot showing NR1H3 transcript expression across major microenvironmental subsets. Dot size indicates the percentage of cells expressing NR1H3, and color intensity indicates the average expression level. **(B)** Violin plot showing inferred NR1H3 transcription factor activity across major microenvironmental subsets, highlighting the strongest activity in Angio-TAMs. **(C)** Bubble plot showing the metabolic profiles of the myeloid subsets using GSVA across canonical metabolic pathways. The dot size corresponds to the relative GSVA score, and the color gradient ranges from low (blue) to high (red) enrichment. **(D)** Lollipop plot ranking top 20 perturbed downstream genes following the *in silico* knockout of *NR1H3*. Genes are ranked by their respective perturbation distance, with dot size indicating fold change and color intensity reflecting the false discovery rate ($-\log_{10}$[FDR]). **(E, F)** Dot plots showing the top enriched terms from GO biological process **(E)** and KEGG pathway **(F)** analyses for the genes affected by NR1H3 perturbation. (**G**) RT-qPCR analysis of *NR1H3* and *APOE* expression in THP-1-derived macrophage-like cells under different treatment conditions. Control cells were transfected with negative-control siRNA, whereas the A431-CM + siNC and A431-CM + si*NR1H3* groups were stimulated with 50% A431-conditioned medium and transfected with negative-control siRNA or siRNA targeting *NR1H3*, respectively (*$p < 0.05$; ** $p < 0.01$; ****$p < 0.0001$). (**H**) Representative images of the Transwell invasion assay showing A431 cell invasion induced by conditioned supernatants collected from the indicated macrophage-like cell treatment groups.

1.2 Supplementary Tables

Supplementary Table 1 Clinical and pathological characteristics of the patients.

Patient ID	Sex	Age	Anatomical Site	Preoperative Diagnosis	Postoperative Diagnosis (Tumor)	Postoperative Diagnosis (Adjacent)	Histological Differentiation
1	Male	88	Cheek	cSCC	cSCC	Normal	Moderately differentiated
2	Female	84	Nose	cSCC	cSCC	Normal	Moderately differentiated
3	Male	67	Head	cSCC	cSCC	Normal	Moderately differentiated

Supplementary Table 2 Differentially expressed marker genes across all identified cell lineages and subpopulations in the cSCC microenvironment.

Supplementary Table 3 Primer and siRNA sequences used in this study.
